# Cholecystocolonic Fistula

**DOI:** 10.7759/cureus.4874

**Published:** 2019-06-10

**Authors:** Summar-un-nisa Abbasi, Dawar B Khan, Kumail Khandwala, Rabail Raza, Wasim A Memon

**Affiliations:** 1 Radiology, Aga Khan University Hospital, Karachi, PAK

**Keywords:** gallbladder, colon, gallstones, cholelithiasis, ileus, cholecystocolonic fistula, ct

## Abstract

Cholecystocolonic fistula (CCF) is a rare complication of gallstone disease with a variable clinical presentation. It is difficult to diagnose CCF pre-operatively despite modern diagnostic and imaging modalities as they are often asymptomatic or incidentally discovered, often peri-operatively. However, management of this uncommon yet important finding is not very well described in the literature. The most common fistula is the cholecystoduodenal fistula, followed by the cholecystocolonic fistula; the cholecystogastric fistula is reportedly the least commonly reported. We report our experience with three cases of cholecystocolonic fistula discovered on imaging which were subsequently confirmed through surgery.

## Introduction

Cholecystocolonic fistula (CCF) results from a communication between the gallbladder and the right side of colon. CCF is usually a late sequela of chronic gallstone disease and is the second most common cholecystoenteric fistula after the cholecystoduodenal fistula. It is discovered in approximately 0.1% of cholecystectomies [1]. Women are more commonly affected than men and it is usually prevalent during the sixth or seventh decade of life. It has been stated that the triad of pneumobilia, chronic diarrhea, and Vitamin K malabsorption is pathognomonic for cholecystocolonic fistula [2]. We report our experience with three cases with variable clinical presentations displaying features suggestive of cholecystocolonic fistula on computed tomography imaging and which were subsequently confirmed by surgery.

## Case presentation

Case 1

A 57-year-old lady came to the ER with complaints of right upper quadrant pain, vomiting, and fever. On clinical examination, the patient was suspected to have a liver abscess and admitted for further management. Laboratory workup showed leukocytosis with a total leukocyte count of 17 x 10^9^/L.

**Figure 1:**
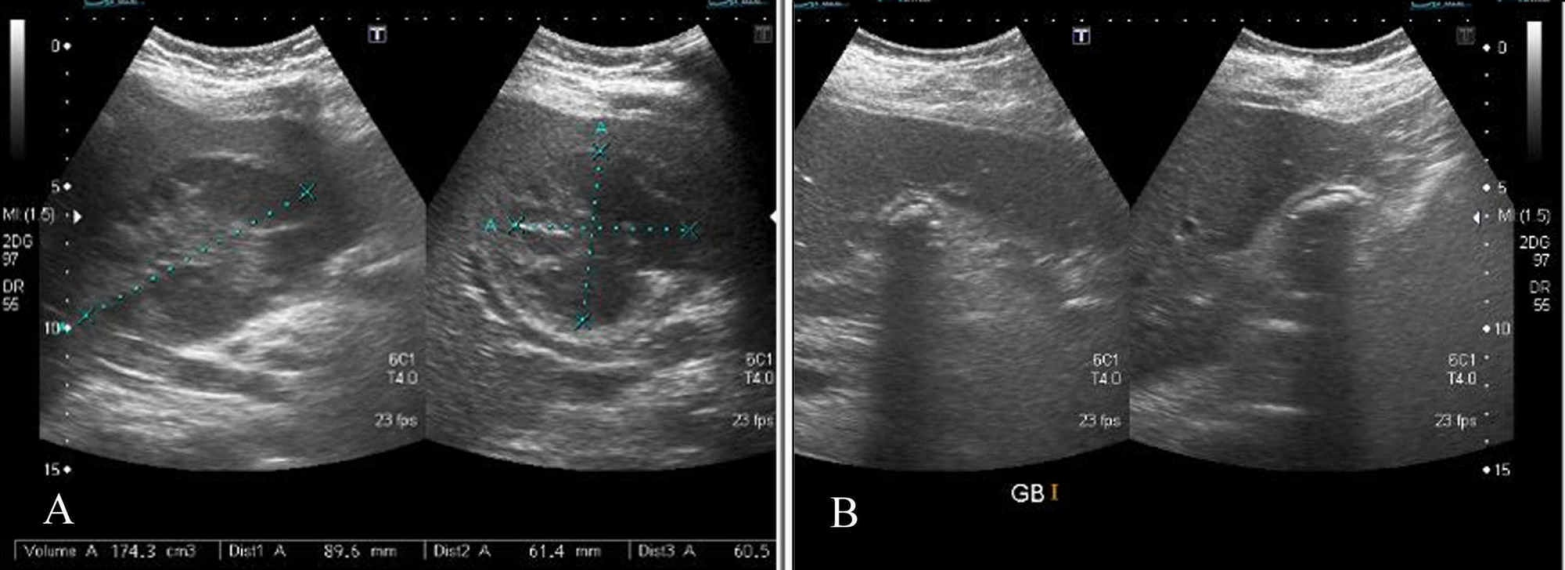
Ultrasound images A) Liver abscess in segment IV, with a volume of approximately 175 cc. B) Contracted gallbladder containing numerous calculi giving wall echo sign.

An ultrasound of the upper abdomen was performed. It showed a large, ill-defined, heterogeneous area without internal vascularity in the right lobe of the liver, predominantly involving segment IV which was assumed to be a liver abscess (Figure 1A). It had a volume of approximately 175 cc. The gallbladder was contracted and contained multiple calculi (Figure 1B).

**Figure 2:**
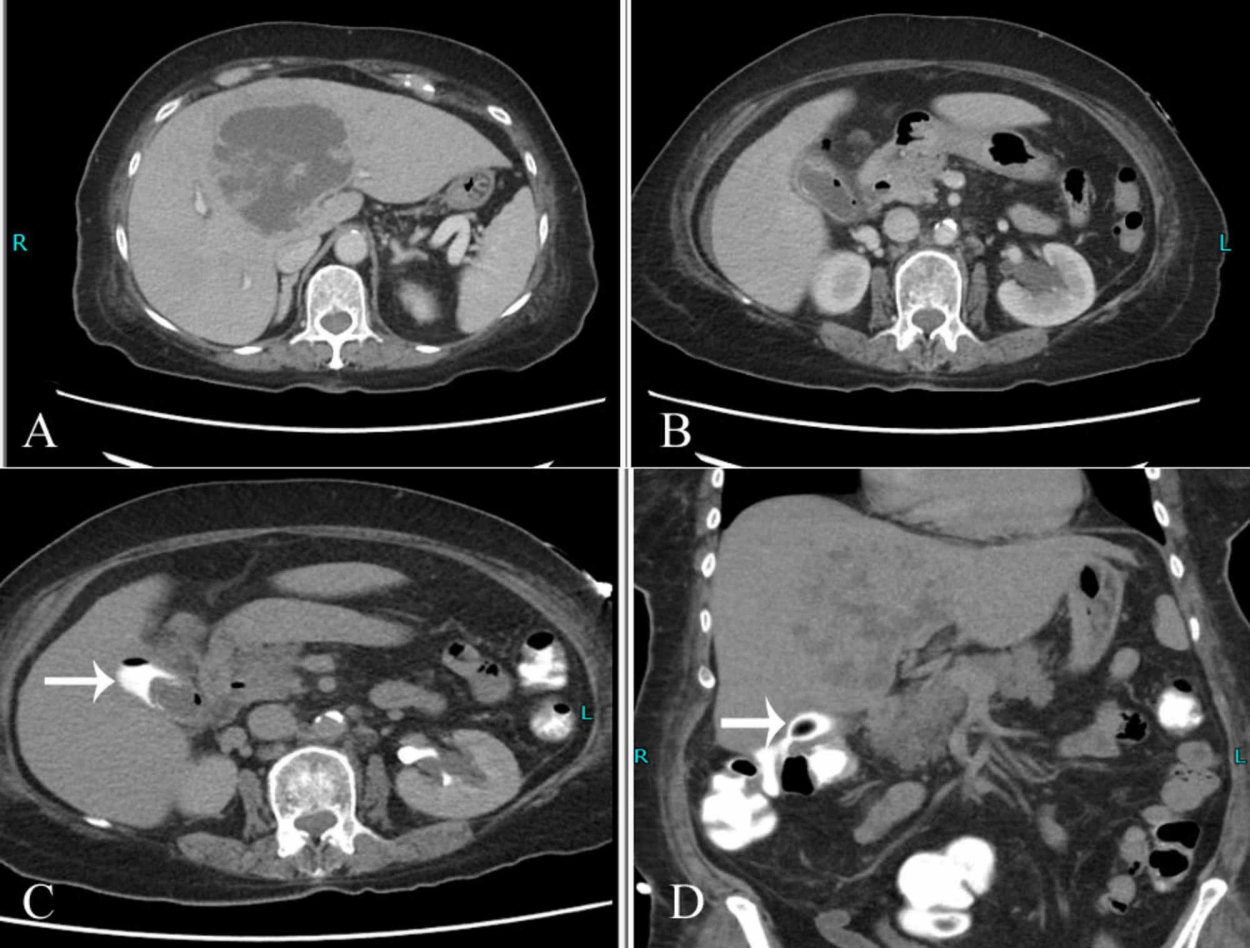
Computed tomography images A) Large necrotic liver abscess noted in segment IV. B) Specks of air seen within the gallbladder lumen. C & D) After rectal contrast administration, opacification of the gallbladder was noted (arrow) confirming the suspicion of cholecystocolonic fistula.

Subsequently, a CT scan was performed to confirm the findings. In addition to the liver abscess, the scan showed air within the gallbladder lumen with fistulous communication between gallbladder and adjacent colon (Figure 2A, 2B). Rectal contrast was also administered which opacified the gallbladder scan, thereby confirming the findings (Figure 2C, 2D).

The patient underwent an exploratory laparotomy, cholecystectomy, and fistula repair. The post-operative gross and histopathological assessment showed an edematous and extremely inflamed gallbladder with dense fibrovascular adhesions and a cholecystocolonic fistula opening into the transverse colon, with a length of less than 1 cm.

Case 2

A 56-year-old female, who was a known case of diabetes mellitus and hypertension, presented with a history of on-and-off fever, vomiting, and upper abdominal pain for three weeks. Her laboratory workup showed raised gamma-glutamyl transferase of 242 IU/L (<38) and alkaline phosphatase of 924 IU/L (45-129) suggesting cholestasis. She had a raised total leukocyte count of 15 x 10^9^/L.

**Figure 3:**
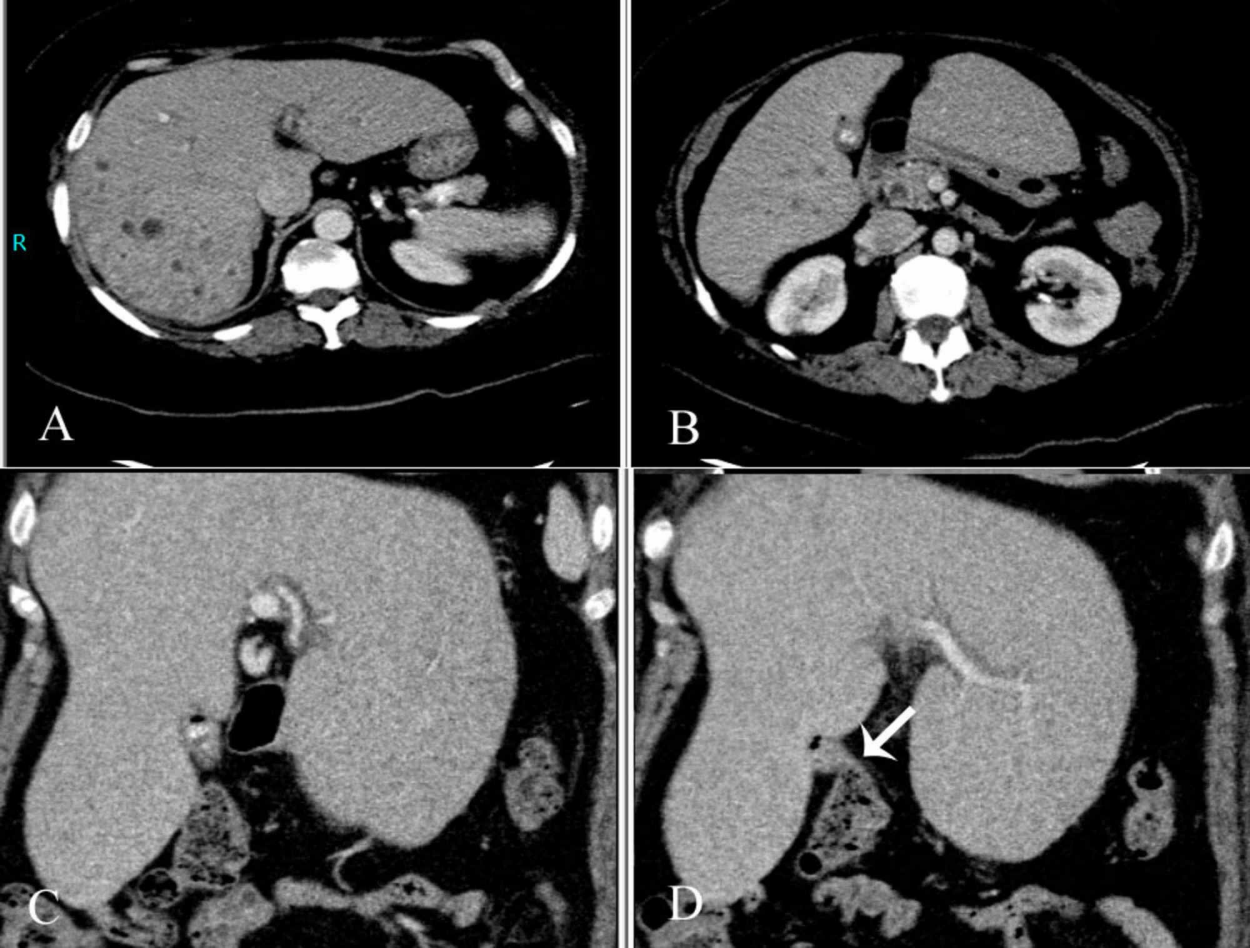
Computed tomography images A & B) Numerous cholangitic abscesses were seen in the right lobe of liver with contracted gallbladder containing calculi and few intraluminal air specks. C & D) Close approximation of hepatic flexure of colon with the gallbladder suggesting a fistulous communication (arrow).

A contrast-enhanced CT scan was performed which revealed cholelithiasis along with few hyperdense calculi in the distal common bile duct (CBD). Mild intrahepatic biliary dilatation was seen, as well as multiple low attenuation lesions in the right lobe of liver with enhancing walls, representing cholangitis abscesses (Figure 3A, 3B). The gallbladder was contracted and contained air specks. It was in close approximation of the hepatic flexure, suggesting cholecystocolonic fistula (Figure 3C, 3D).

Gastroenterology and general surgery were conducted. The patient underwent an exploratory laparotomy with cholecystectomy and CBD exploration. A fistulous tract was identified between the hepatic flexure of the colon and fundus of the gallbladder during the surgery which was repaired. Multiple calculi were seen in the distal CBD along with the shrunken gallbladder with thickened walls, suggestive of chronic cholecystitis.

Case 3:

A 34-year-old male patient presented to the ER with complaints of abdominal pain, jaundice, and vomiting for one week. He had a history of a small soft-tissue mass near gallbladder fundus for which an ultrasound-guided biopsy was done two years prior. Histopathology revealed signs of inflammation without any malignancy.

Current laboratory investigations showed deranged liver function tests (Table 1).

**Table 1:** Liver function tests GGT: Gamma-glutamyl transferase SGPT: Serum Glutamate-Pyruvate Transaminase

Parameter	Result	Normal Range
Total Bilirubin	7.4	(0.1-1.2 mg/dL)
Direct Bilirubin	6.0	(0-0.2 mg/dL)
Indirect Bilirubin	1.4	(0.1-0.8 IU/L)
GGT	182	<55 IU/L
SGPT	205	<45 IU/L

**Figure 4:**
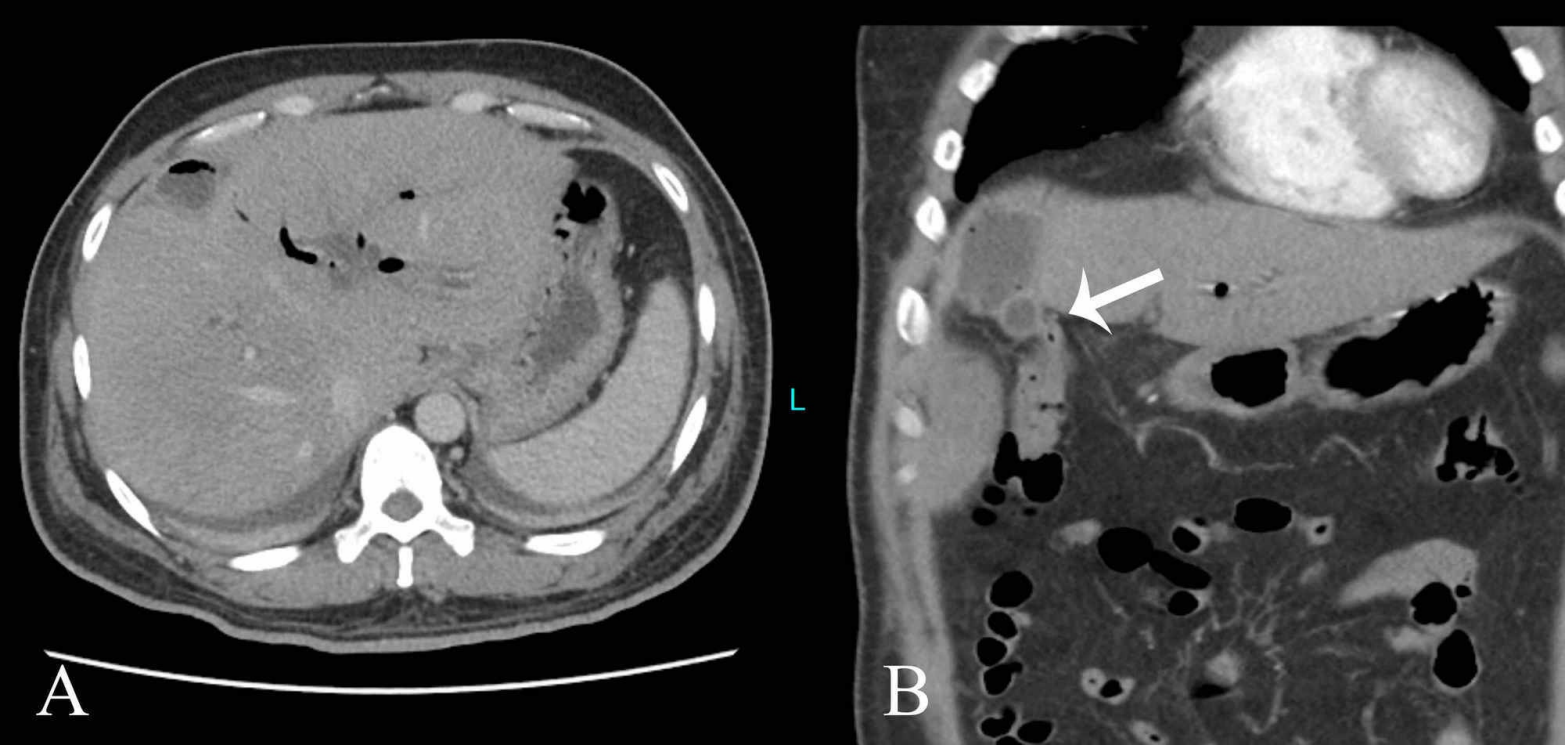
Computed tomography images A) Pneumobilia seen in the intrahepatic biliary channels and air specks noted within the gallbladder lumen. B) Fistulous communication was demonstrated between the gallbladder and colon (arrow).

A contrast-enhanced CT scan was performed that showed mild intrahepatic biliary dilatation with pneumobilia. Air was also identified within the gallbladder lumen (Figure 4A). The hepatic flexure was in close proximity to the gallbladder with possible communication, so suspicion of cholecystocolonic fistula was raised (Figure 4B).

Percutaneous transhepatic biliary tube placement was done to drain the biliary system, and after initial stabilizaton and resuscitation, the patient underwent an exploratory laparotomy which confirmed the findings of a small (1-1.5 cm) cholecystocolonic fistula, pus-filled gallbladder indicating empyema, and a dilated CBD with a large distal CBD calculus (not seen on CT).

## Discussion

The first cholecystoenteric fistula was described by Courvoisier in the 1890s. Cholecystocolonic fistula is usually a late sequela of gallstone disease with an incidence of one in 1000 cholecystectomies [3-5]. Because of a lack of common presenting symptoms and rare occurrence, it usually difficult to diagnose this condition pre-operatively and therefore it is not a frequently reported abnormality.

CCF occurs mainly as a result of inflammation in the gallbladder due to cholecystitis. Glenn et al. [3] suggest that acute cholecystitis with obstruction of the cystic duct leads to the formation of adhesions with the adjacent organs, including the colon. Recurrent inflammation results in ulceration and ischemia of the wall of the gallbladder, thereby causing erosion and eventually fistula formation. However, other associated conditions like cancer, trauma, amebic infections, peptic ulcer disease, and diverticulitis have also been implicated in the etiology [6-7]. 

Patients with CCF usually have a variable clinical presentation and more often asymptomatic. When symptomatic, patients generally present with diarrhea, abdominal pain, jaundice, fever, nausea, vomiting, steatorrhea, and weight loss. The combination of pneumobilia, chronic diarrhea, and vitamin K malabsorption has been proposed as a pathognomonic triad for cholecystocolonic fistula [1]. However, this classic clinical triad was not observed in either of our patients. CCF alters the enterohepatic circulation, leading to a malabsorption syndrome with loss of water and electrolytes from the large bowel which results in diarrhea and weight loss. Rarely, CCF can result in stone impaction in the region of rectosigmoid and can cause large-bowel obstruction, in contrast to small bowel obstruction by gallstone ileus [7]. In our series, two out of three cases had CCF with associated gallstone disease, biliary obstruction, and liver abscesses, suggesting that the fistula was an incidental finding discovered due to the investigation of other hepatobiliary diseases. However, none of them had an intestinal obstruction. 

Pre-operative studies have infrequently been able to make a definite diagnosis of CCF. Imaging may include ultrasound, CT scan, MRI, endoscopic retrograde cholangiopancreatography (ERCP), and barium enema, but a diagnosis is often made perioperatively [4-5]. When suspected intraoperatively, the diagnosis can be confirmed with a cholangiogram. However, ERCP has been considered to be the most accurate diagnostic modality of CCF preoperatively by some authors [7]. Pneumobilia has been considered to be associated with CCF especially if the gallbladder is shrunken and in close proximity to bowel on CT. Only one patient in our series had pneumobilia; however, air in the gallbladder was present in all three cases suggesting that this is a more sensitive finding on CT for raising a strong suspicion of fistula based on the anatomical proximity with the bowel. 

Undiagnosed CCF on preoperative imaging may pose a problem for the surgeon, who is often forced to convert the elective laparoscopic cholecystectomy to a complex open procedure that may involve adhesiolysis and colonic resection. Therefore, the ideal treatment for a suspected biliary-enteric fistula should be an open cholecystectomy with the closure of the fistula. A very small number of reports [4-5, 8-9, 10] have explored laparoscopic management of CCF. Although the authors have advocated the practicality of the laparoscopic approach, they have also raised concerns over long operating times and the need for converting into an open procedure due to complications like iatrogenic colonic perforation. CCFs are often associated with several other complications such as acute cholangitis, biliary peritonitis, and biliary cirrhosis, with an overall mortality rate ranging between 10% to 15% [1]. In our case series, all three patients underwent complex open procedures and no laparoscopic treatment was sought.

## Conclusions

In conclusion, while the cholecystocolonic fistula is a rare entity, it is one that radiologists should be aware of and it should be carefully reported if present in conjunction with other hepatobiliary abnormalities. Hence, failure to identify these fistulas during surgery can lead to complications, such as iatrogenic perforation of the colon and resultant fecal peritonitis, which could lead to severe sepsis.
